# Green tea aqueous extract (GTAE) prevents high‐fat diet‐induced obesity by activating fat browning

**DOI:** 10.1002/fsn3.2580

**Published:** 2021-10-06

**Authors:** Jie Li, Qiyang Chen, Xiuming Zhai, Dan Wang, Yujia Hou, Min Tang

**Affiliations:** ^1^ Research Institute of Tea Chongqing Academy of Agricultural Sciences Chongqing China; ^2^ College of Horticulture and Landscape Architecture Southwest University Chongqing China

**Keywords:** BAT, browning, green tea, Ing‐WAT, low‐grade systemic inflammation, obesity

## Abstract

Adipose browning leads to increased energy expenditure and reduced adiposity and has, therefore, become an attractive therapeutic strategy for obesity. In this study, we elucidated the effect of green tea aqueous extract (GTAE) on the browning of inguinal white adipose tissue (Ing‐WAT) and brown adipose tissue (BAT) in high‐fat diet (HFD)‐fed mice. The main phytochemical components identified in GTAE through high‐performance liquid chromatography (HPLC) included (−)‐gallocatechin, (−)‐epigallocatechin, (−)‐catechin, (−)‐epigallocatechin‐3‐gallate, caffeine, (−)‐epicatechin, (−)‐gallocatechin gallate, and (−)‐epicatechin‐3‐gallate. Daily supplementation with 1% GTAE for 12 weeks markedly reduced bodyweight gain, systemic inflammation, oxidative stress, and improved insulin resistance. Additionally, histological analysis revealed that dietary supplementation with 1% GTAE reversed HFD‐induced adipocyte size and hepatic steatosis. These effects were associated with activation of browning in the Ing‐WAT and BAT, which mediate systemic metabolic dysfunction in HFD‐fed mice. Taken together, our data support the use of GTAE, a natural product, for the attenuation of obesity through the activation of fat browning.

## Graphical abstract

The present study found that mice fed a high‐fat diet showed significantly lower weight gain upon GTAE administration than their control counterparts. In addition, GTAE ameliorated the high‐fat diet‐induced hepatic steatosis, insulin resistance, and low‐systematic inflammatory. These effects were associated with browning activation in the Ing‐WAT and BAT.

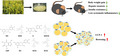

## 1 INTRODUCTION

The global prevalence of overweight and obesity has increased markedly, and obesity has become a serious health concern (Afshin et al., 2017). Moreover, obesity is a risk factor for the development of many diseases, including type‐2 diabetes, nonalcoholic fatty liver disease, cardiovascular disease, and several cancer types among others (Hruby & Hu, 2015; Ren et al., 2021). Emerging evidence suggests the occurrence of pathogenic events in the development and progression of obesity‐associated comorbidities, including low‐grade systemic inflammation, oxidative stress, and insulin resistance (Hernandez‐Bautista et al., 2019; Romeo et al., 2012). Therefore, it is critical to investigate the physiological mechanisms during the occurrence and development of obesity.

In mammals, brown adipose tissue (BAT) is metabolically highly active and dissipates energy as heat through nonshivering thermogenesis (Cannon & Nedergaard, 2004). In addition, BAT is a multilocular organ that contains a relatively large number of mitochondria that produce heat via uncoupling of the respiratory chain (Poekes et al., 2015). BAT maintains temperature in newborn infants and small mammals (McMillan & White, 2015). Recent studies have demonstrated that adults also possess metabolically active brown fat or “beige adipocyte” (browning of white adipose tissues [WATs]) (Cypess et al., 2009; Ozguven et al., 2016; Wang & Seale, 2016). Beige adipocytes are morphologically and functionally similar to BAT, which increases energy expenditure and improves lipidemia and glucose metabolism (Kim & Plutzky, 2016). Thus, activation of BAT activity or induction of beige adipocytes represents an attractive approach to prevent diet‐induced obesity and related metabolic diseases. Clinically, drug therapy and surgery are effective treatments for obesity, but they often have side effects. Recently, many phytochemicals from natural products have been reported to regulate the adipocyte life cycle. These phytochemicals induce fat‐browning activity, lipolysis, and trans‐differentiation of white to brown‐like adipocytes (Azhar et al., 2016; Okla et al., 2017).

Green tea (*Camellia sinensis* [L.] O. Kuntze) is one of the most common and popular beverages in the world, and owing to its production process, green tea retains abundant tea polyphenols, especially epigallocatechin gallate (EGCG) (Wang et al., 2014). Several studies have demonstrated that green tea and green tea‐derived polyphenols exert preventive effects against obesity (Ma et al., 2020; Okla et al., 2017; Zhu et al., 2020). However, the effects of green tea aqueous extract (GTAE) on adipose browning have not been fully elucidated. In the present study, we examined whether long‐term supplementation with GTAE decreased HFD‐induced obesity in mice. Our data indicate that GTAE reduces obesity and improves obesity‐associated comorbidities in treated mice. These effects are mediated by the regulation of systemic metabolic dysfunction and brown‐like adipocyte formation.

## 2 MATERIALS AND METHODS

### 2.1 Reagents

(−)‐gallocatechin, (−)‐epigallocatechin, (−)‐catechin, (−)‐epigallocatechin‐3‐gallate, caffeine, (−)‐epicatechin, (−)‐gallocatechin gallate, and (−)‐epicatechin‐3‐gallate were purchased from Biopurify Phytochemicals Ltd. (Chengdu, China) at a purity >95% according to the manufacturer's specifications. Antibodies against nuclear factor‐kappa light‐chain enhancer of activated B cells (NF‐κB), uncoupling protein‐1 (UCP‐1), and β‐tubulin were obtained from Affinity Biosciences Inc. All other reagents were purchased from Sigma‐Aldrich unless otherwise noted.

### 2.2 Preparation of GTAE

Green tea (Yucha 4#) was obtained from the Research Institute of Tea (Chongqing Academy of Agricultural Sciences). Dried green tea powder was extracted using a material‐liquid ratio of 1:20 (w/v) at 100℃ for 20 min, and then the residue was extracted for another 10 min. The extracted liquids were combined and filtered, evaporated (55℃) using a rotary evaporator, and finally lyophilized to a powder form. The final powder was stored at −80℃ until further use.

### 2.3 HPLC analysis

GTAE powder (25 mg) was dissolved in methanol (25 ml) and then filtered through a 0.22 µm filter prior to HPLC. An Agilent 1200 series HPLC system was used for quantitative analysis of the GTAE powder. The constituents were separated at 278 nm using an Agilent C18 column (250 × 4.6 mm, 5 µm) under control conditions (column temperature, 30℃; injection volume, 10 µl). The mobile phase consisted of methanol (A) and water (B) at a flow rate of 0.8 ml/min. The gradient elution protocol used was as follows: 0–20 min, 80%–50% A; 20–25 min, 50%–75% A.

### 2.4 Animals and experimental design

Eight‐week‐old male C57BL/6 mice (22–24 g) were purchased from the Experimental Animal Center of Chongqing Medical University (Certification: SCXK 2018–0003). All animal protocols were approved by the Southwest University Experimental Animal Ethics Committee (approval no: 0,004,649). Mice were housed (three‐four animals per cage) in a humidity‐controlled environment (12 h light/12 h dark cycles, 22 ± 2℃). Following 1‐week of adaption, 40 mice (four groups, *n* = 10) were treated as follows: normal chow diet (NCD), high‐fat diet (HFD, 40% of calories from fat), NCD plus 1% (w/w) GTAE (NCD +1% GTAE), and HFD plus 1% (w/w) GTAE (HFD +1% GTAE) for 12 weeks. All mice had access to drinking water and food ad libitum. Bodyweight and food intake were monitored every other day.

### 2.5 Biochemical analysis

Intraperitoneal glucose tolerance test (IGTT) and insulin tolerance test (ITT) were conducted on 12 h‐fasting mice on the last day of the study period. Tail vein blood was used to determine blood glucose levels using a Roche glucometer (Accu‐Chek Active, Roche, Mannheim, Germany) at time intervals of t = 15, 30, 45, 60, 90, and 120 min after intraperitoneal injection of 0.5 U insulin/kg (Sigma) and 2 g glucose/kg (Sinopharm Chemical Reagent Co. Ltd.). Homeostatic model assessment of insulin resistance (HOMA‐IR = fasting blood glucose [mmol/L] × insulin [mIU/L]/22.5) was used to estimate insulin resistance (Matthews et al., 1985). At the end of the experimental period, the mice were anesthetized with 20% urethane and blood samples were obtained via cardiac puncture. Serum samples were collected by centrifugation at 3000 × *g* for 15 min. Serum alanine aminotransferase (ALT), aspartate aminotransferase (AST), total cholesterol (TC), triacylglycerol (TG), glucose, insulin, tumor necrosis factor‐α (TNF‐α), interleukin‐2 (IL‐2), lipopolysaccharide (LPS), and liver TG and TC were measured using commercial ELISA kits from Sino Best Biological Technology Co. Ltd.

### 2.6 Histological analysis

Ing‐WAT, BAT, and liver tissues were fixed using 4% paraformaldehyde overnight and then embedded in paraffin. Tissue sections (4 µm thick) were stained with hematoxylin and eosin (H&E), Oil Red O, or immunostained for UCP‐1. The size of Ing‐ WAT was analyzed using an ImageJ software, as described previously (Chou et al., 2018).

### 2.7 Immunoblot analysis

Total protein was extracted from the liver using Total Protein Extraction kit (Beyotime Biotech) according to the manufacturer's instructions. Protein samples were electrophoresed using sodium dodecyl sulfate‐polyacrylamide gel electrophoresis (SDS‐PAGE) on a 10% gel and then electro‐transferred for 1.5–2 h onto polyvinylidene fluoride (PVDF) membranes (Millipore). The membranes were blocked with 5% nonfat milk for 1.5 h and then blotted with primary antibodies (NF‐κB and β‐actin) overnight at 4℃. After washing with Tris‐buffered saline Tween‐20 (TBST), the membranes were probed with antirabbit IgG (1:5000, Proteintech, SA00001‐2) for 1 h. Protein expression was visualized using a gel image analysis system (Tanon‐3500R) and quantified by densitometry.

### 2.8 Quantitative real‐time qPCR

Total RNA was extracted from the liver, Ing‐WAT, and BAT using TRIzol reagent (Invitrogen). cDNA was synthesized using a Reverse Transcription cDNA Synthesis kit (Tiangen Biotech Co. Ltd.). The qPCR was performed using NovoStart®SYBR qPCR SuperMix plus (Annoron) following the manufacturer's recommendations. The primers used in this study are listed in Table S1. A reference gene (*β‐actin*) was used to normalize the amount of template.

### 2.9 Statistical analysis

All results are presented as mean ± standard deviation (SD). Statistical differences were analyzed using one‐way analysis of variance with Duncan's multiple range test using SPSS 18.0.

## 3 RESULTS

### 3.1 Phytochemical components in GTAE

**FIGURE 1:**
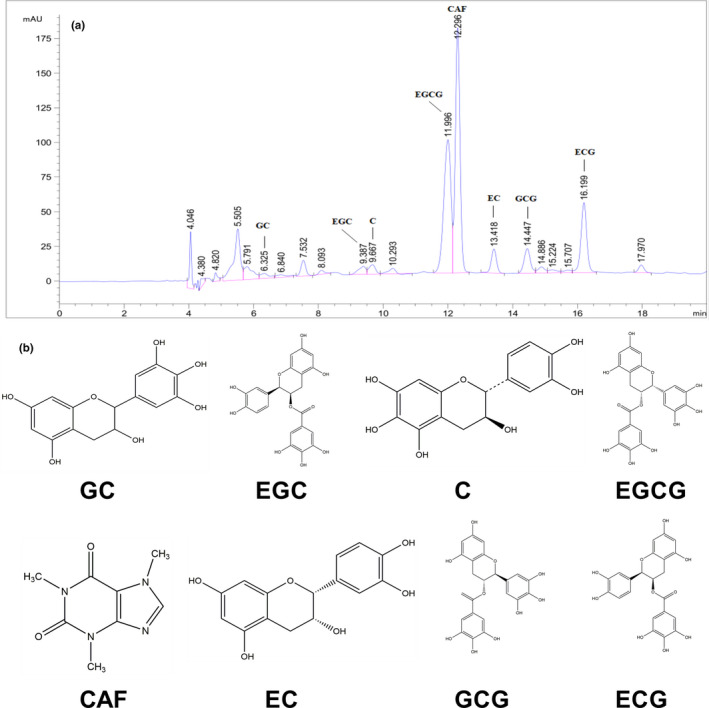
The main phytochemical components in green tea aqueous extract (GTAE). (a) HPLC chromatogram; (b) Chemical structure of (−)‐gallocatechin (GC), (−)‐epigallocatechin (EGC), (−)‐catechin (c), (−)‐ epigallocatechin‐3‐gallate (EGCG), caffeine (CAF), (−)‐epicatechin (EC), (−)‐gallocatechin gallate (GCG), and (−)‐epicatechin‐3‐gallate (ECG)

The constituents of GTAE were analyzed using HPLC. Catechins and caffeine content in GTAE are shown in Figure 1 and Table 1. (−)‐gallocatechin (GC, 28.93 ± 1.95 mg/g), (−)‐epigallocatechin (EGC, 100.16 ± 6.87 mg/g), (−)‐catechin (C, 6.30 ± 0.35 mg/g), (−)‐epigallocatechin‐3‐gallate (EGCG, 126.82 ± 5.22 mg/g), caffeine (CAF, 50.01 ± 1.17 mg/g), (−)‐epicatechin (EC, 9.43 ± 0.70 mg/g), (−)‐gallocatechin gallate (GCG, 19.79 ± 0.74 mg/g), and (−)‐epicatechin‐3‐gallate (ECG, 24.40 ± 0.82 mg/g) were detected in GTAE. The total amount of phytochemical components in GTAE was approximately 365.54 ± 14.87 mg/g.

**TABLE 1:** Catechins and caffeine content in GTAE

Compound	GTAE (mg/g powder)
(−)‐gallocatechin (GC)	28.93 ± 1.95
(−)‐epigallocatechin (EGC)	100.16 ± 6.87
(−)‐catechin (C)	6.30 ± 0.35
(−)‐epigallocatechin−3‐gallate (EGCG)	126.82 ± 5.22
caffeine (CAF)	50.01 ± 1.17
(−)‐epicatechin (EC)	9.43 ± 0.70
(−)‐gallocatechin gallate (GCG)	19.79 ± 0.74
(−)‐epicatechin−3‐gallate (ECG)	24.4 ± 0.82

Note

GTAE, green tea aqueous extract.

### 3.2 GTAE prevents HFD‐induced obesity

**FIGURE 2:**
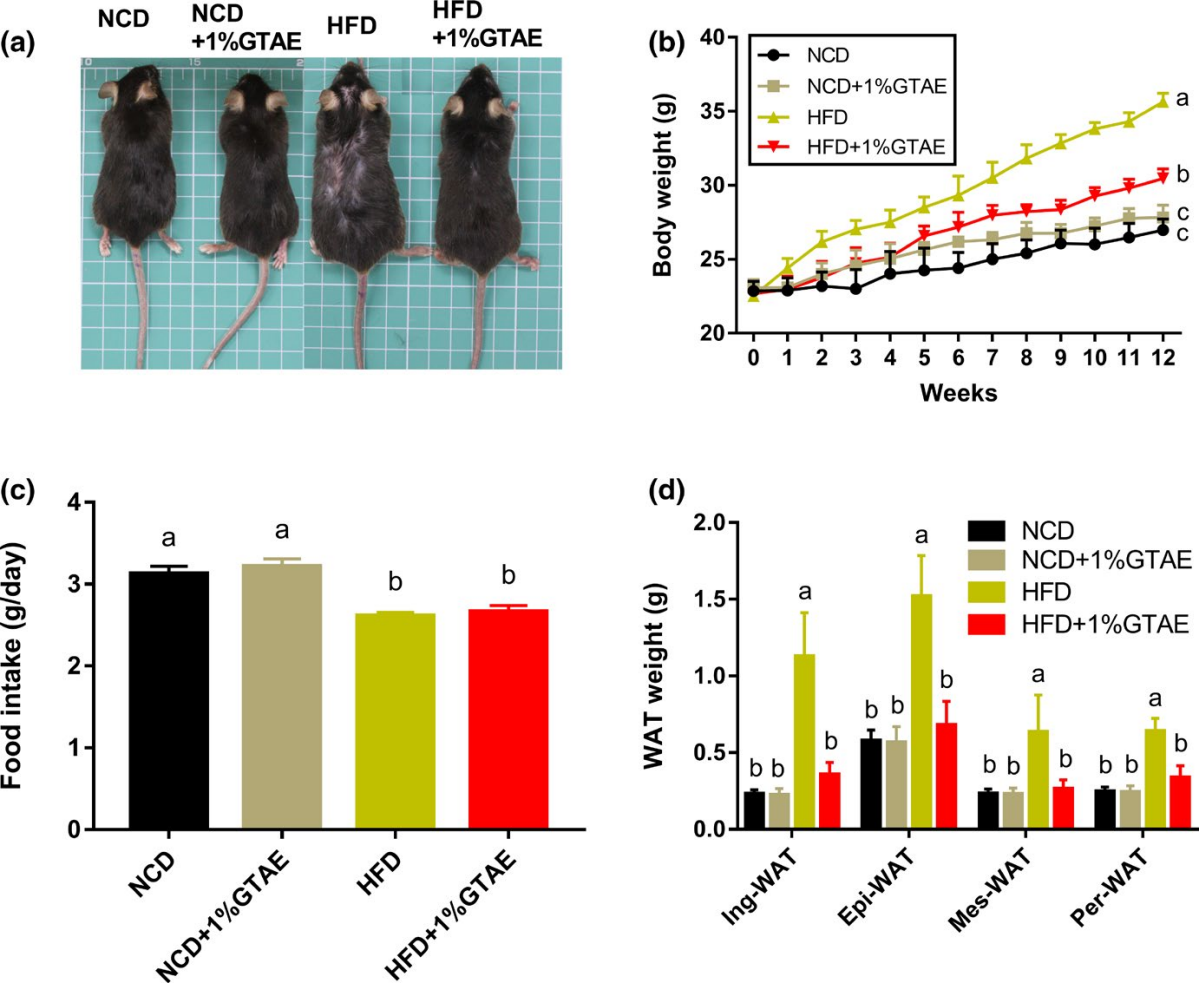
Effects of GTAE on body weight in C57BL/6 mice fed high‐fat diet. (a) Phenotypes of representative mice from each group. (b) Body weight was monitored weekly for 12 weeks. (c) Average food intake in each group. (d) Weights of the four types of white adipose tissue (WAT), including inguinal white adipose tissue (Ing‐WAT), epididymal white adipose tissue (Epi‐WAT), mesenteric white adipose tissue (Mes‐WAT), and perirenal white adipose tissue (Per‐WAT). Values with different letters (a, b, and c) are significantly different (*p* < .05) as determined by Duncan's multiple range test

To examine the effects of GTAE supplementation on the bodyweight of mice, 8‐week‐old male C57BL/6 mice were fed either an NCD, NCD +1% GTAE, HFD, or HFD +1% GTAE for 12 weeks. As expected, HFD significantly increased the bodyweight of mice (Figure 2a), which was 1.32‐fold higher than that of the NCD group (26.97 ± 0.43 g). Supplementation with 1% GTAE markedly decreased the bodyweight of HFD‐fed mice (Figure 2b). During the treatment, food intake was not affected by 1% GTAE in mice fed with NCD or HFD (Figure 2c). In addition, analyses of the four main types of WATs including inguinal white adipose tissue (Ing‐WAT), perirenal white adipose tissue (Per‐WAT), mesenteric white adipose tissue (Mes‐WAT), and epididymal white adipose tissue (Epi‐WAT) revealed that consistent with results of the bodyweight, 1% GTAE significantly inhibited the increase in WAT (*p* < .05).

### 3.3 GTAE improves dyslipidemia and insulin resistance

**FIGURE 3:**
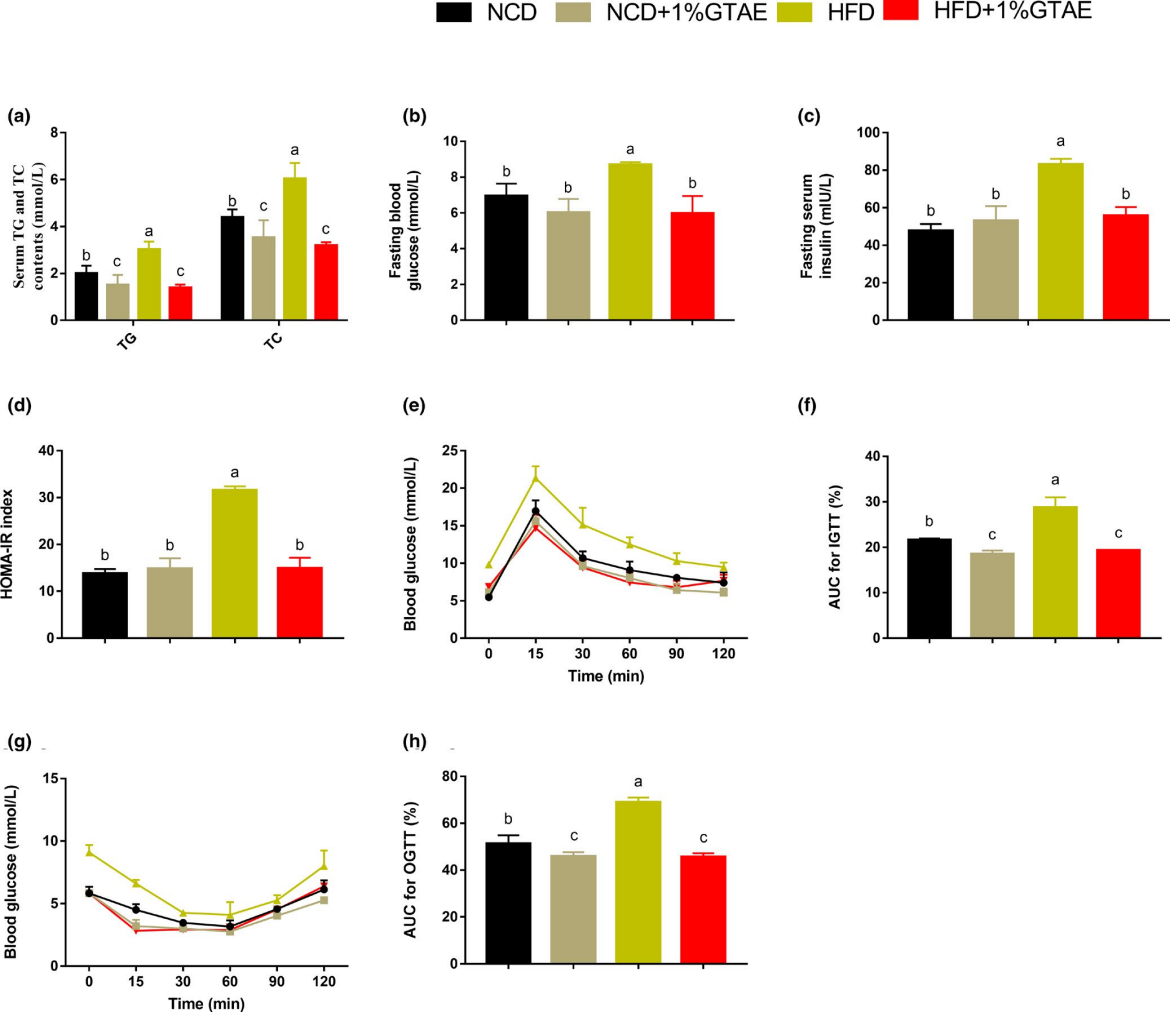
Effects of GTAE on insulin resistance in high‐fat diet (HFD)‐fed mice. (a) Serum concentrations of triacylglycerol (TG) and total cholesterol (TC). (b‐c) Fasting blood glucose and insulin levels. (d) Homeostatic model assessment ‐ insulin resistance (HOMA‐IR) index. (e, f) Intraperitoneal glucose tolerance test (IGTT) and corresponding area under the curve (AUC). (g, h) Insulin tolerance test (ITT) and corresponding area under the curve (AUC). Values with different letters (a, b, and c) are significantly different (*p* < .05) as determined by Duncan's multiple range test

To determine whether GTAE has protective effects against hyperinsulinemia and hyperlipidemia, we analyzed serum lipid concentrations. As shown in Figure 3a, supplementation with GTAE markedly decreased serum TG and TC levels in HFD‐fed mice. Obesity is strongly associated with glucose tolerance and insulin resistance. Therefore, we evaluated fasting blood glucose and insulin levels and the corresponding HOMA‐IR. HFD induced higher fasting blood glucose and insulin concentrations compared to NCD, whereas GTAE reversed these effects (Figure 3b–d). Moreover, the corresponding HOMA‐IR index was also markedly reduced in the HFD +1% GTAE group. Furthermore, intraperitoneal glucose tolerance test (IGTT), insulin tolerance test (ITT), and area under the curve (AUC) confirmed that GTAE had a positive effect by improving HFD‐induced insulin resistance (Figure 3e–h). These results indicate that GTAE plays a positive role in reducing HFD‐induced dyslipidemia and insulin resistance.

### 3.4 GTAE inhibits HFD‐induced liver steatosis and systemic low‐grade inflammation, and improves antioxidant capacity

**FIGURE 4:**
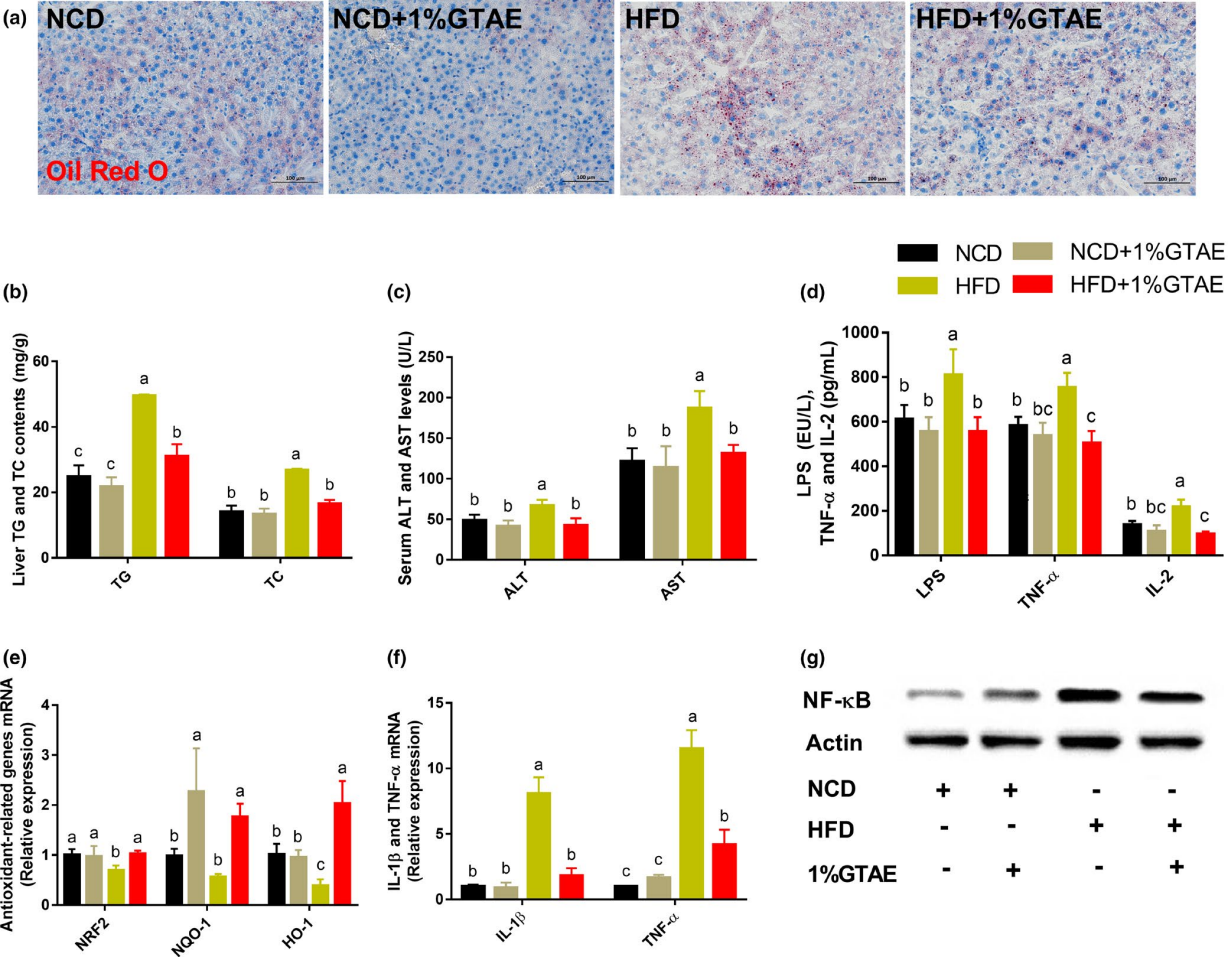
Effects of GTAE on hepatic steatosis, systemic inflammation, and antioxidant activity in high‐fat diet (HFD)‐fed mice. (a) Liver tissue sections stained with Oil Red O (scale: 100 μm). (b) TG and TC content in each treatment group. (c) Serum alanine aminotransferase (ALT) and aspartate aminotransferase (AST) levels. (d) Serum endotoxin (lipopolysaccharide, LPS), tumor necrosis factor‐α (TNF‐α), and interleukin‐2 (IL‐2) concentrations in each group. (e, f) Expression of antioxidant‐ (NRF2, NQO‐1, and HO‐1) and inflammation‐related genes (IL‐1β, TNF‐α) (g) Expression of NFκB protein in the liver. Values with different letters (a, b, and c) are significantly different (*p* < .05) as determined by Duncan's multiple range test

Lipid accumulation is closely correlated with hepatic steatosis. Therefore, we assessed the effects of GTAE on hepatic steatosis. GTAE administration inhibited lipid accumulation in the liver and prevented liver steatosis, as evidenced by the results of Oil Red O staining and liver TG and TC levels (Figure 4a,b). In addition, lower plasma alanine aminotransferase (ALT) and aspartate aminotransferase (AST) levels and higher mRNA expression of antioxidant‐related genes (*NRF2*, *NQO‐1*, and *HO‐1*) indicated that GTAE exhibited protective effects against HFD‐induced liver damage (Figure 4c,e). We further investigated the impact of GTAE on lipopolysaccharide (LPS, endotoxin) and inflammatory cytokines. GTAE markedly reduced the serum levels of LPS, tumor necrosis factor‐α (TNF‐α), interleukin‐2 (IL‐2), and inflammation‐related genes (*TNF‐α* and *IL‐1β*) in the hepatic tissues (Figure 4d,f). As shown in Figure 4g, GTAE restored the level of HFD‐induced NF‐κB protein. These findings imply that GTAE exhibits robust efficacy against liver steatosis and ameliorates systemic low‐grade inflammation.

### 3.5 GTAE stimulates browning of Ing‐WAT and BAT

**FIGURE 5:**
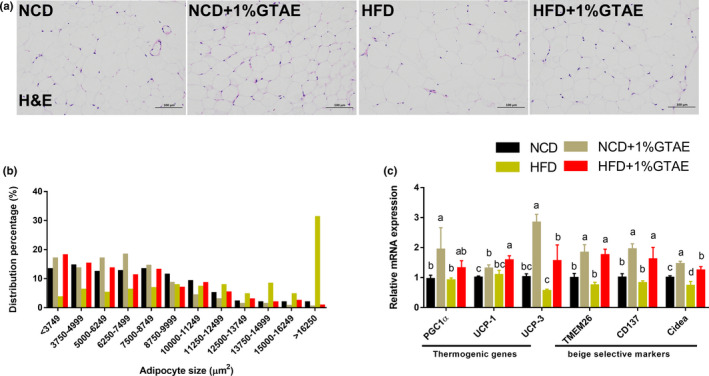
Effects of GTAE on adipocyte hypertrophy and WAT browning. (a) H&E staining of inguinal white adipose tissue (Ing‐WAT) sections (scale: 100 μm). (b) Inguinal adipocyte size distribution in each group. (c) mRNA levels of thermogenic genes and beige adipocyte‐selective markers in Ing‐WAT. Values with different letters (a, b, c, and d) are significantly different (*p* < .05) as determined by Duncan's multiple range test

**FIGURE 6:**
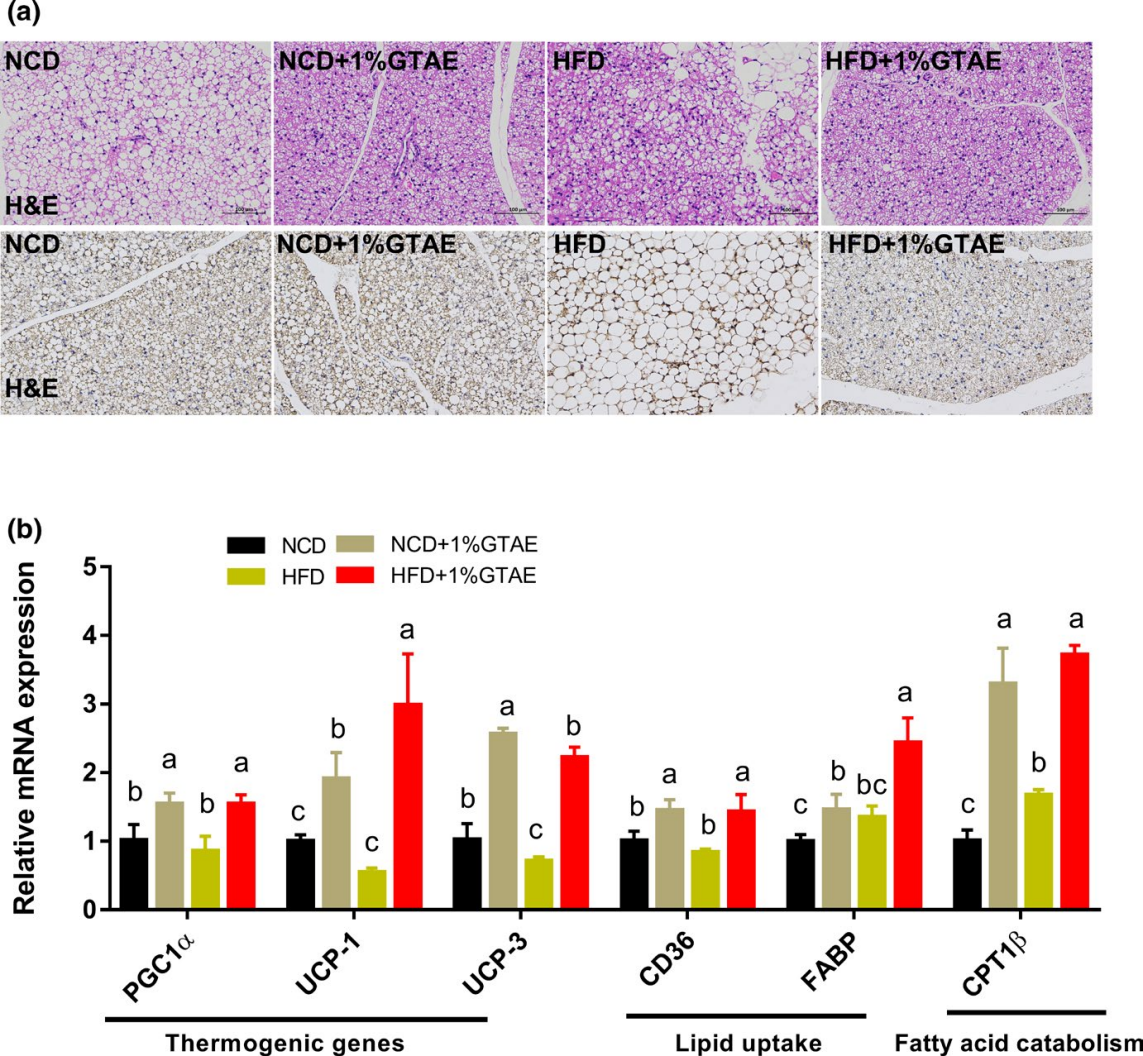
Effects of GTAE on brown adipose tissue (BAT) browning. (a) H&E and uncoupling protein‐1 (UCP1) immunofluorescence staining of representative BAT sections (scale: 100 μm). (c) mRNA levels of genes related to thermogenics, lipid uptake, and fatty acid catabolism in BAT. Values with different letters (a, b, and c) are significantly different (*p* < .05) as determined by Duncan's multiple range test

Stimulating the development of beige adipocytes in WAT (also called browning) may ameliorate the adverse effects of WAT and further improve the management of obesity (Bartelt & Heeren, 2014). As shown in Figure 5a,b, HFD‐fed mice displayed increased adipocyte size in the white fat compared to NCD‐fed mice, whereas treatment with GTAE restored the normal size. Moreover, thermogenic genes (*PGC1α*, *UCP‐1*, and *UCP‐3*) and beige‐selective markers (*TMEM26*, *CD137*, and *Cidea*) were upregulated by GTAE, indicating that GTAE promoted the browning program (Figure 5c). Next, we examined the effects of GTAE on BAT, which is involved in adaptive nonshivering thermogenesis. As shown in Figure 6a,c, HFD‐fed mice displayed larger multilocular adipocytes, whereas GTAE reversed this to normal levels and upregulated the expression of UCP‐1, a thermogenic protein. In addition, GTAE also increased the expression of genes involved in thermogenesis (*PGC1α*, *UCP‐1*, and *UCP‐3*), lipid transport (*CD36* and fatty acid‐binding proteins [*FABP*]), and fatty acid catabolism (*CPT1β*). These results demonstrate that GTAE stimulates the thermogenesis of Ing‐WAT and BAT in HFD‐induced mice.

## 4 DISCUSSION

The rise in obesity is a major global public health concern (Lim et al., 2012; Morgen & Sorensen, 2014). Obesity results from an imbalance between energy intake and expenditure. Therefore, mechanisms involved in increased energy expenditure may regulate weight homeostasis and combat obesity. Emerging studies have shown that green tea (*C. sinensis*) potentially enhances lipolysis and energy expenditure to prevent obesity (Bolin et al., 2020; Neyrinck et al., 2017). Herein, we report that GTAE markedly inhibited bodyweight gain in HFD‐fed mice. Moreover, GTAE ameliorated HFD‐induced insulin resistance, liver steatosis, oxidative damage, and systemic low‐grade inflammation. Importantly, GTAE enhanced thermogenic gene expression, further promoting the browning of Ing‐WAT and BAT.

As reported in previous studies, green tea has potential antiobesity properties (Dey et al., 2019; Dinh et al., 2019; Westerterp‐Plantenga, 2010). These properties are attributed to several bioactive compounds, such as EC, EGC, ECG, and EGCG (Hayat et al., 2015; Li et al., 2020). In the present study, we detected eight major phytochemicals in GTAE, including GC, EGC, C, EGCG, CAF, EC, GCG, and ECG, of which EGCG was present at the highest concentration (Table 1). A previous study showed that 0.5% tea catechins suppressed HFD‐induced bodyweight gain, epididymal, retroperitoneal, and perirenal fat accumulation, and the development of hyperinsulinemia and hyperleptinemia in mice (Murase et al., 2002). Additionally, EGCG (1 mg kg^‐1^ day^‐1^) also increased lean mass and improved glucose tolerance in HFD‐fed mice (Chen et al., 2009). Our results showed that 1% GTAE markedly reduced bodyweight and fat mass and improved dyslipidemia and insulin resistance compared to the control HFD group. Thus, our findings suggest that GTAE may be a promising therapeutic agent against diet‐induced obesity.

Low‐grade chronic inflammation in obesity is a major risk factor for the development of many metabolic disorders (Asghar & Sheikh, 2017). Consistent with a previous study, GTAE reduced serum endotoxins, mainly produced by gram‐negative bacteria (Li et al., 2018). In our study, GTAE decreased the circulating levels of systemic low‐grade inflammatory markers (TNF‐α and IL‐2) and reversed NF‐κB protein expression in the livers of HFD‐fed mice. As previously described, green tea extract and green tea catechins ameliorate inflammatory responses and oxidative stress by suppressing NF‐κB activation (Li et al., 2017; Marinovic et al., 2015). Additionally, GTAE stimulates the expression of *Nrf2* and its target genes *NQO‐1* and *HO‐1* in the liver, indicating that GTAE protects hepatocytes against oxidative stress potentially via the NRF2 pathway (Gao et al., 2018; Sampath et al., 2017). Since NRF2 activates fatty acid oxidation‐related genes and further improves the lipid blood profile, this may explain the mechanism through which GTAE attenuates lipid content in the liver (Vasileva et al., 2020).

Browning activation of brown‐in‐white (brite) adipose cells within white adipose pads induce uncoupling and heat production capacities, enabling enhanced lipolysis (Cohen & Spiegelman, 2015; Sidossis et al., 2015). A previous study showed that human subcutaneous WAT is transformed into an energy‐dissipating tissue (Sidossis et al., 2015). In our study, we observed that GTAE markedly reduced HFD‐induced Ing‐WAT hypertrophy. Previous studies have shown that antiadiposity is associated with reduced expression of several inflammatory markers (Cunha et al., 2013; Sae‐Tan et al., 2015), which is consistent with our results. A recent study reported that green tea may involve the PPARγ/FGF21/AMPK/UCP‐1 pathway, promoting the induction of thermogenic cells by reprogramming the initial steps of adipocyte commitment (Bolin, Sousa‐Filho, dos Santos, et al., 2020). In this study, we show that GTAE induces browning markers in Ing‐WAT. Correspondingly, PGC‐1α, a transcriptional master coregulator of mitochondrial biogenesis, was highly induced by GTAE. This transcriptional activation is involved in regulating the promoter activity of UCP‐1 (Bostrom et al., 2012). Several beige‐type‐specific markers, including *TMEM26*, *CD137*, and *Cidea*, have been identified in mice and humans (Bartelt & Heeren, 2014). These genes were activated by GTAE, indicative of changes in the Ing‐WAT. Taken together, these results suggest that GTAE induces the formation and activity of energy‐dissipating tissues (in terms of increased metabolism and heat production).

Accumulating evidence indicates that BAT secretes factors involved in the regulation of systemic metabolism (Wang et al., 2015). This emerging concept provides a physiologically significant link between BAT and systemic metabolism. Obesity leads to the whitening of BAT, characterized by mitochondrial dysfunction and lipid droplet accumulation (Shimizu et al., 2014). In our study, we observed that the number of smaller multilocular adipocytes increased in the BAT of GTAE‐treated mice. In addition, BAT is a powerful metabolic organ that metabolizes plasma glucose and triglycerides in mice (Bartelt & Heeren, 2012). Within brown adipocytes, fatty acids are internalized by CD36, while non‐esterified fatty acids are trafficked to mitochondria using FABPs to generate heat (Bartelt & Heeren, 2012). In the present study, GTAE elevated the expression of *CD36* and *FABP*, indicating that GTAE enhances lipid transport. Previous studies have shown that green tea increases the expression of BAT *UCP‐1* (Neyrinck et al., 2017; Nomura et al., 2008). The activation of *UCP‐1* and other thermogenic genes in brown adipocytes is crucial for heat production (Bostrom et al., 2012). Consistent with these results, GTAE significantly induced the expression of these genes, which may be attributed to the high concentration of EGCG (Neyrinck et al., 2017).

In conclusion, our study demonstrates that GTAE prevents HFD‐induced obesity, as evidenced by the inhibition of bodyweight gain, fat mass, and WAT hypertrophy. In addition, GTAE improves insulin resistance, liver steatosis, and systemic low‐grade inflammation, and induces browning of Ing‐WAT and BAT. Data from our study contribute valuable information for understanding the function of GTAE in the prevention of obesity.

## CONFLICTS OF INTEREST

The authors declare that there is no conflicts of interest.

## AUTHOR CONTRIBUTIONS

**Jie Li:** Conceptualization (equal); Formal analysis (equal); Funding acquisition (equal); Methodology (equal); Writing‐original draft (equal). **Qiyang Chen:** Formal analysis (equal); Investigation (equal); Methodology (equal); Writing‐review & editing (equal). **Xiuming Zhai:** Investigation (equal). **Dan Wang:** Investigation (equal); Writing‐review & editing (equal). **Yujia Hou:** Conceptualization (equal). **Min Tang:** Conceptualization (equal); Writing‐review & editing (equal).

## Supporting information

Table S1
