# Supplementary material for: Green tea aqueous extract (GTAE) prevents high‐fat diet‐induced obesity by activating fat browning
Source: Food Sci Nutr. 2021 Oct 6;9(12):6548–58. doi: 10.1002/fsn3.2580 (PMC8645728; doi:10.1002/fsn3.2580)
Supplement: Supplementary file 1 — Table S1 [file FSN3-9-6548-s001.doc]

| **Table S1 Real-time PCR primer pairs.** | | |
| --- | --- | --- |
| gene | Forward primer (5'-3') | Reverse primer (5'-3') |
| NRF2 | GCCTCCAAAGGATGTCAATCA | GCCTCACCTCTGCTGCAAGTA |
| NQO1 | TGGCGTAGTTGAATGATGTCTT | TTCGGTATTACGATCCTCCCT |
| HO-1 | CCACATTGGACAGAGTTCACAG | CCTCACAGATGGCGTCACTTC |
| TNF-α | ATGGATCTCAAAGACAACCAACTAG | ACGGCAGAGAGGAGGTTGACTT |
| IL-1β | TCGTGCTGTCGGACCCATAT | GGTTCTCCTTGTACAAAGCTCATG |
| UCP1 | TAAGCCGGCTGAGATCTTGT | GGCCTCTACGACTCAGTCCA |
| UCP3 | ATGAGTTTTGCCTCCATTCG | GGCGTATCATGGCTTGAAAT |
| PGC1α | GGAGCCGTGACCACTGACA | TGGTTTGCTGCATGGTTCTG |
| FABP | AGCATCATAACCCTAGATGGCG | CATAACACATTCCACCACCAGC |
| CD36 | GGAACTGTGGGCTCATTGC | CATGAGAATGCCTCCAAACAC |
| CPT1β | GGCACCTCTTCTGCCTTTAC | TTTGGGTCAAACATGCAGAT |
| TMEM26 | GAAACCAGTATTGCAGCACCCAAT | AATATTAGCAGGAGTGTTTGGTGGA |
| CD137 | GTCGACCCTGGACGAACTGCTCT | CCTCTGGAGTCACAGAAATGGTGGTA |
| Cidea | ATCACAACTGGCCTGGTTACG | TACTACCCGGTGTCCATTTCT |
| β-actin | AACCGTGAAAAGATGACCCAGAT | CACAGCCTGGATGGCTACGT |
